# Let-7 family regulates HaCaT cell proliferation and apoptosis via the ΔNp63/PI3K/AKT pathway

**DOI:** 10.1515/med-2024-0925

**Published:** 2024-03-23

**Authors:** Min Li, Yi Ding, Tayier Tuersong, Long Chen, Mei-Lin Zhang, Tian Li, Shu-Mei Feng, Qiong Guo

**Affiliations:** Department of Histology and Embryology, School of Basic Medical Sciences, Xinjiang Medical University, Urumqi, 830000, Xinjiang, China; Department of Human Anatomy, School of Basic Medical Sciences, Xinjiang Second Medical College, Karamay, 834000, Xinjiang, China; Department of Pharmacy, The Second Affiliated Hospital of Xinjiang Medical University, Urumqi, 830000, Xinjiang, China; Functional Center, School of Basic Medical Sciences, Xinjiang Medical University, Urumqi, 830000, Xinjiang, China; Xinjiang Urumqi City Center Blood Station, Urumqi, 830000, Xinjiang, China; Key Laboratory of Xinjiang Uygur Autonomous Region, Laboratory of Molecular Biology of Endemic Diseases, Urumqi, 830000, Xinjiang, China; Department of Histology and Embryology, School of Basic Medical Sciences, Xinjiang Medical University, No. 567 Suntech North Road, Shuimogou District, Urumqi, 830000, Xinjiang, China

**Keywords:** fetal skin, let-7 family, ΔNp63, scar-free skin healing, phosphoinositide 3-kinase-protein kinase B signaling

## Abstract

We evaluated the expression profiles of differentially expressed miRNAs (DEmiRNAs) involved in human fetal skin development via high-throughput sequencing to explore the expression difference and the regulatory role of miRNA in different stages of fetal skin development. Analysis of expression profiles of miRNAs involved collecting embryo samples via high-throughput sequencing, then bioinformatics analyses were performed to validate DEmiRNAs. A total of 363 miRNAs were differentially expressed during the early and mid-pregnancy of development, and upregulated DEmiRNAs were mainly concentrated in the let-7 family. The transfection of let-7b-5p slowed down HaCaT cell proliferation and promoted apoptosis, as evidenced by the cell counting kit-8 assay, quantitative real-time polymerase chain reaction, and flow cytometry. The double luciferin reporter assay also confirmed let-7b-5p and ΔNp63 downregulation through the combination with the 3ʹ-untranslated region of ΔNp63. Moreover, treatment with a let-7b-5p inhibitor upregulated ΔNp63 and activated the phosphoinositide 3-kinase (PI3K)-protein kinase B (AKT) signaling pathway. The let-7b-5p caused a converse effect on HaCaT cells because of Np63 upregulation. Let-7b-5p regulates skin development by targeting ΔNp63 via PI3K-AKT signaling, contributing to future studies on skin development and clinical scar-free healing.

## 1 Introduction

The repair of skin wounds after skin injury is vital for the structural and functional recovery of the skin. However, adult skin wounds often scar when healing [1]. Studies have shown that wounds in embryonic skin heal without scarring and that the skin structure and regeneration-associated appendages are reconstructed [2–4]. Recently, some studies have found that microRNAs (miRNAs) play a vital regulatory role in skin repair.

As small non-coding RNAs, miRNAs can influence gene expression and cell function. Recent investigations have established that miRNAs, mostly through annealing to the 3′-untranslated region (UTR) of mRNAs, negatively influence gene expression at the post-transcriptional stage [5]. Certain miRNAs reportedly are crucial in skin aging [6,7] and skin tumor formation [8–10]. Furthermore, miRNAs functions are connected to the development and differentiation of epithelial cells [11]. For instance, miR-31 can enhance keratinocyte proliferation and migration ability [12]. In addition, mir-13 can also accelerate the re-epithelialization of healing skin tissue [13]. Although certain studies have shown that gene therapy may represent an effective approach to enhance wound healing [14–16], no effective treatment for preventing scar formation in adult skin is currently available [17]. Macrophages play a key role in the resolution of inflammation and tissue repair, and the let-7 family is involved in macrophage polarization. Studies have shown that the miRNA let-7 is associated with cell differentiation and that the expression of let-7b-5p is significantly enhanced in M2 macrophages. Let-7 can also target signal transducer and activator of transcription 3 in macrophages to inhibit LPS-induced inflammation and exert anti-inflammatory effects [15]. In many tissues and conditions, mirs from the let-7 family appear to be particularly involved in signaling growth and stress responses in multiple cell types, especially after extracellular injury [18]. Many let-7 mirs have significant effects on the regulation of inflammatory processes. Let-7i has been implicated in wound repair in many cell types, in part due to the interaction with progesterone [19]. Dermal fibroblasts also expressed elevated levels of aging biomarkers affecting telomere maintenance and various stages of the cell life cycle, such as let-7, miR-23a-3p, 34a-5p, miR-125a, miR-181a-5p, and miR-221/222-3p. Macrophages are key coordinators of inflammation, fibrosis, and wound repair. Macrophages coordinate the process of wound healing by shifting from a predominantly pro-inflammatory (M1-like phenotype) (present early after injury) to an anti-inflammatory (M2-like phenotype) [19]. M2-type macrophages promote angiogenesis, ECM repair, anti-inflammatory cytokine release, and resolution of inflammation. Therefore, it is speculated that let-7 family may affect skin healing by regulating the polarization of macrophages. The different polarization states of macrophages have important effects on the fibrotic process, and the regulation of the let-7 family may help to maintain the proper balance of macrophages and prevent excessive fibrotic responses. Therefore, investigating the miRNAs that regulate skin development may provide new research clues for developing gene therapy for scar-free healing after skin trauma.

Current research, including sequencing studies, has focused on cell sequencing or animal skin tissue sequencing. However, the fetal and adult wound healing processes [20] as well as skin development from the embryonic to adult stages [21] substantially differ between humans and laboratory animals. Human fetal skin morphogenesis implies continuous skin stratification and horizontal expansion [22]. Notably, human fetal skin stratification begins at week 11 [23]. In the current study, we investigated the expression mode, function, and mechanism of action of miRNAs during skin development by analyzing the expression patterns of miRNAs in human fetal samples at the beginning of skin development to screen for differentially expressed miRNAs (DEmiRNAs) that play an essential regulatory function in fetal skin development.

## 2 Materials and methods

### 2.1 Human fetal skin samples

Human fetal skin tissues were collected at five hospitals from seven fetuses following spontaneous abortion. Samples were assigned to group 0 according to the fetal age (<11 weeks of gestation; *n* = 3) and group 1 (>11 weeks of gestation; *n* = 4). Hospital ethics committees approved this experiment (Urumqi, Xinjiang, China), and 100% of the women gave us the informed consent.


**Ethics approval and consent to participate:** The study was approved by the First Affiliated Hospital of Xinjiang Medical University (No. 20160218-113). Written informed consent was obtained from all individuals included in this study.

### 2.2 Small RNA sequencing

Total RNA was isolated from each sample as the starting material to build the miRNA library. All the RIN (RNA integrity number) values for the total RNA reached 7–8. After the sample passed the quality test, the miRNA library construction and high-throughput sequencing were completed by Personalbio (Shangai, China). Different index tags were selected and created by Illumina (NEB, USA), followed by second-generation sequencing by next-generation sequencing using paired-end reads using the Illumina HiSeq sequencing platform.

### 2.3 Network analyst analysis

The Limma package was used to evaluate DEmiRNAs between groups 0 and 1. DEmiRNAs were characterized as miRNAs with log2 (fold change) >1 and a *P*-value 0.01 after correction. By applying NetworkAnalyst 3.0 (https://www.networkanalyst.ca/), a volcano plot and principal component analysis (PCA) of the DEmiRNAs were carried out [24].

### 2.4 Target mRNA enrichment analyses

We used the TargetScan (http://www.targetscan.org), miRanda (http://www.microrna.org), and RNA22 (https://cm.jefferson.edu/rna22/) tools to determine the DEmiRNAs’ potential binding targets based on the intersections provided by the three tools above. The potential targets were then used by DAVID (https://david.ncifcrf.gov/) for gene ontology (GO) and Kyoto Encyclopedia of Genes and Genomes (KEGG) functional enrichment analysis.

### 2.5 HaCaT cell culture

The human keratinocyte cell line HaCaT, an immortalized epithelial cell line from the skin tissue of *Homo sapiens* [21] (China Center for Type Culture Collection, Wuhan, China), was kept up in Dulbecco’s Modified Eagle Medium supplemented with 10% fetal bovine serum (Thermo Fisher Scientific, Waltham, MA, USA) and 1% penicillin–streptomycin (Thermo Fisher Scientific).

### 2.6 Transient transfection

Cells were transfected with miRNA mimics, inhibitors, simulated negative control (NC) (30–50% confluence), or inhibitor NC (purchased from Ruibo Bio, Guangzhou, China). Cells were used for functional and mechanistic analyses 48 h after transfection.

### 2.7 Cell counting kit-8 (CCK-8) assays

We used the CCK-8 kit (Bioss, Beijing, China) in accordance with the manufacturer’s instructions to assess cell proliferation. About 10 L of the CCK-8 solution was briefly implemented to each culture well. After that, cells were cultivated at 37°C for 2 h. After that, a microplate meter (ReadMax 1200; Thermo Fisher Scientific) was used to test the cell’s absorbance at 450 nm.

### 2.8 Flow cytometry

Propidium iodide (Thermo Fisher Scientific) and Annexin V-fluorescein isothiocyanate (BD Biosciences, Franklin Lake, NJ, USA) were utilized to identify cells that had died. A flow cytometer was used to measure a FACSCanto II system (BD Biosciences).

### 2.9 Quantitative real-time PCR (qPCR)

HaCaT cells’ total DNA was isolated using TRIzol (Thermo Fisher Scientific), and cDNA was produced using the ReverTra Ace qPCR RT Kit (TransScript, Beijing, China). In Table 1, used primers are displayed.

**Table 1:** Primer sequence details of real time PCR

Primer name	Orientation	Sequence (5′–3′)
*β-actin*	Forward	CAACTTGATGTATGAAGGCTTTGGT
*β-actin*	Reverse	ACTTTTATTGGTCTCAAGTCAGTGTACAG
*BCL-XL*	Forward	ACATCCCAGCTTCACATAACCC
*BCL-XL*	Reverse	CCATCCCGAAAGAGTTCATTCAC
*BIM*	Forward	TAAGTTCTGAGTGTGACCGAGA
*BIM*	Reverse	GCTCTGTCTGTAGGGAGGTAGG
*BAX*	Forward	CCCGAGAGGTCTTTTTCCGAG
*BAX*	Reverse	CCAGCCCATGATGGTTCTGAT
*BAK*	Forward	ATGGTCACCTTACCTCTGCAA
*BAK*	Reverse	TCATAGCGTCGGTTGATGTCG
*ΔNp63*	Forward	AGCAGTTGTGTTGGAGGGATGAAC
*ΔNp63*	Reverse	TCCGCCTTCCTGTCTCTTCCTG
*U6*	Forward	CTCGCTTCGGCAGCACA
*hsa-let-7b-5p*	Forward	UGAGGUAGUAGGUUGUGUGGUU
*hsa-let-7c-5p*	Forward	UGAGGUAGUAGGUUGUAUGGUU
*hsa-let-7i-5p*	Forward	UGAGGUAGUAGUUUGUGCUGUU
*ribosomal RNA*	Reverse	Common Reverse primer in kit

### 2.10 Lentiviral overexpression

ΔNp63-overexpressing lentivirus, constructed and packed by HanBio (Shanghai, China), was used in accordance with the manufacturer’s instructions to transfect HaCaT cells.

### 2.11 Western blotting

HaCaT cells were obtained, and total proteins were extracted with standard extraction reagents (Thermo Fisher Scientific), including protease and phosphatase inhibitors (Merck, Darmstadt, Germany). SDS-polyacrylamide gel electrophoresis was used to separate the extracted proteins, which were then electrophoretically transferred to nitrocellulose membranes (Millipore, Burlington, MA, USA). Membranes were then blocked for 2 h at room temperature (23–25°C) with 5% skim milk. Membranes were treated with rabbit antibodies against phosphorylated protein kinase B (AKT; p-AKT), phosphoinositide 3-kinase (PI3K), ΔNp63, mechanistic target of rapamycin (mTOR), and phosphorylated mTOR (p-mTOR) (all purchased from Abcam, Cambridge, UK) for 2 h at room temperature. In the end, membranes were developed using Proteinsimple FluorChem E (ProteinSimple, San Jose, CA, USA) reagent, and protein concentration analysis was conducted by ImageJ software (National Institutes of Health, Bethesda, MD, USA).

### 2.12 Luciferase reporter assays

The wild-type (WT) and mutant (MT) target sites at the ΔNp63 3′-UTR were extracted via PCR and then cloned into a pmirGLO vector (Hanbio Biotechnology). Subsequently, 293 cells (1 × 10^4^ cells/well) were inoculated on 12-well plates. Using Lipofectamine 2000 (Thermo Fisher Scientific), miR-7b-5p mimics or NC were transfected into WT or MT Np63 3′-UTR constructs. The luciferase assay system was used to treat the transfected cells after 48 h, and the LB9507 system was used to quantify the luciferase activity.

### 2.13 Statistical analysis

To create statistical maps, GraphPad Prism Version 8.0.1 software (GraphPad Software, San Diego, CA, USA) was utilized. Western blots were analyzed in grayscale with ImageJ, and three independent replications of each experiment were conducted. A *P* value of 0.05 was regarded as statistically significant when comparing groups in bar graphs using an independent sample *t*-test. The mean ± standard deviation were used to express the data.

## 3 Results

### 3.1 Identification of DEmiRNAs

**Figure 1:**
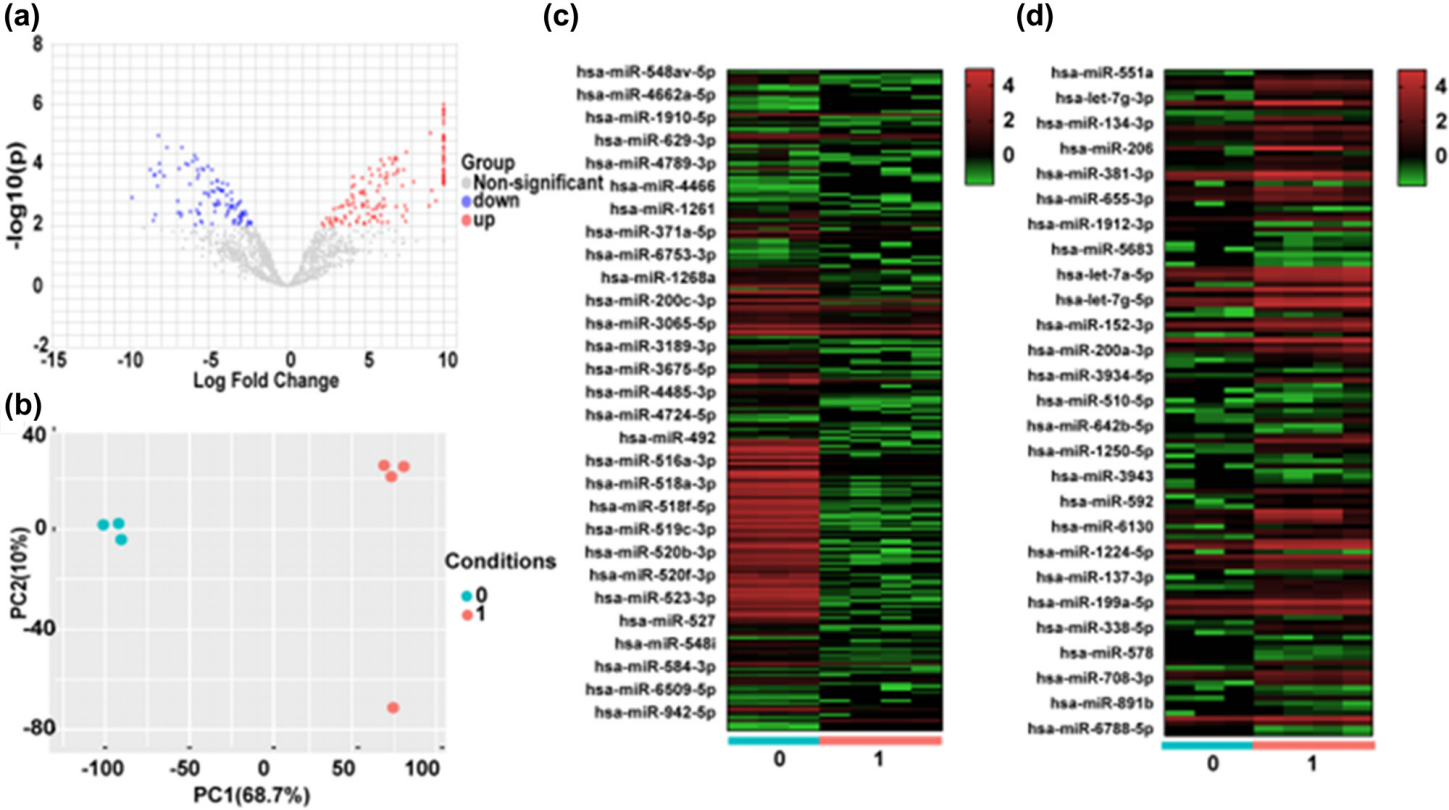
DEmiRNAs in skin samples (|log2(fold change)|>1 and adjusted *P*-value < 0.01) between group 0 (<11 weeks of gestation) and group 1 (>11 weeks of gestation). (a) Volcano plot of DEmiRNAs. Red, blue, and gray spots indicate upregulated, downregulated, and normal expression, respectively, at >11 weeks of gestation. (b) PCA of the miRNA expression in skin samples. (c) Heatmap of downregulated DEmiRNAs. (d) Heatmap of upregulated DEmiRNAs. Rows represent different miRNAs. Columns represent the three samples in group 0 followed by the four samples in group 1. Red and green represent high and low expression of the miRNAs, respectively.

Gene expression data (based on high-throughput sequencing) obtained from skin tissues were subjected to a NetworkAnalyst analysis to identify DEmiRNAs. The PCA showed that the expression profiles of miRNAs in skin samples could be used to distinguish between the two groups (<11 and >11 weeks of gestation) (Figure 1a). In total, 363 miRNAs were identified as DEmiRNAs, and the volcano plot showed that 231 and 132 DEmiRNAs were upregulated and downregulated, respectively, at >11 weeks of gestation (Figure 1b). Each individual skin sample’s DEmiRNA expression was represented with a heatmap (Figure 1c).

### 3.2 GO and KEGG functional analysis

**Figure 2:**
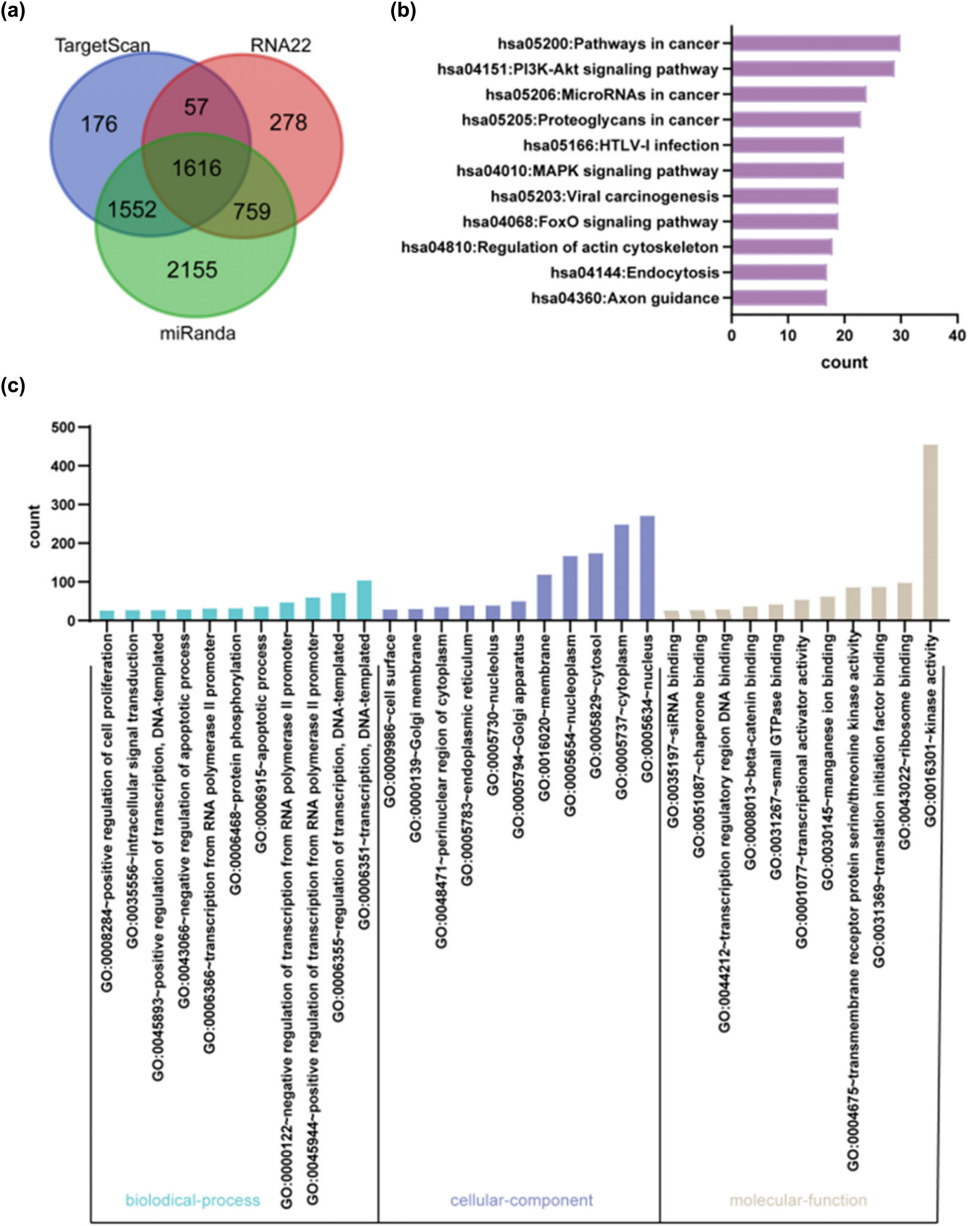
Functional analysis of DEmiRNAs. (a) Venn diagram was predicted by target mRNAs. (b) Histogram of numbers of target mRNAs associated with each of the top ranked KEGG pathways. (c) Histogram of numbers of target mRNAs associated with each of the top ranked GO terms.

We predicted the target mRNAs of the DEmiRNAs and put them to GO analysis to uncover the probable roles of the DEmiRNAs in fetal skin samples. A collection of the target mRNA results predicted using the three databases is presented in Figure 2a. Figure 2c shows the number of target mRNAs associated with each of the top 11 enriched phrases (*P* < 0.05) in the three GO categories (biological processes, cellular components, and molecular functions) The results suggest that the target mRNAs were mostly enriched in “regulation of cell proliferation,” “negative regulation of apoptosis,” and “transcription.” Additionally, KEGG pathway analysis revealed that the PI3K-Akt signaling pathway and cancer development-related pathways were the most important (Figure 2b).

**Figure 3:**
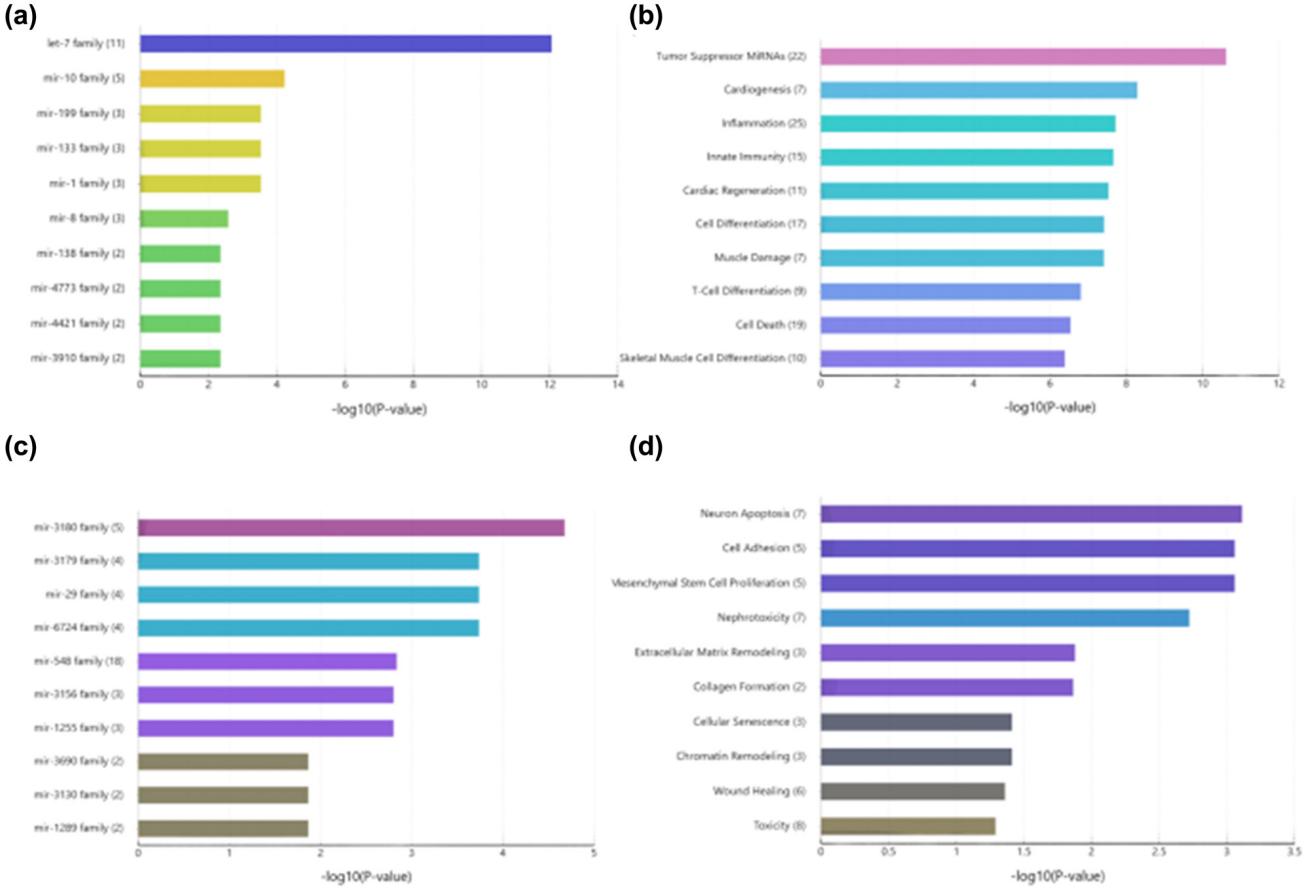
Enrichment analysis of DEmiRNAs. (a) Upregulate the miRNAs family clustering analysis. (b) Upregulated miRNAs function analysis. (c) Downregulate the miRNAs family clustering analysis. (d) Downregulate miRNAs function analysis.

To verify the accuracy of the predictions and further reduce the screening range, enrichment analysis was performed using TAM 2.0 online tools for annotation analysis of miRNAs. We found that upregulated DEmiRNAs in skin samples during the fetal period were mainly enriched in the let-7 family (Figure 3a). miRNAs significantly involved in cell function were identified via miRNA functional analysis (Figure 3b). Family clustering analysis of miRNAs in fetal skin samples showed that downregulated DEmiRNAs were mainly enriched in the miR-3180 family (Figure 3c); miRNAs were relatively more involved in apoptosis, as per the results of functional analyses, in addition to cell adhesion and wound healing (Figure 3d). In summary, these records showed that the let-7 family might be associated with skin development.

### 3.3 Let-7 family affects HaCaT cell proliferation

**Figure 4:**
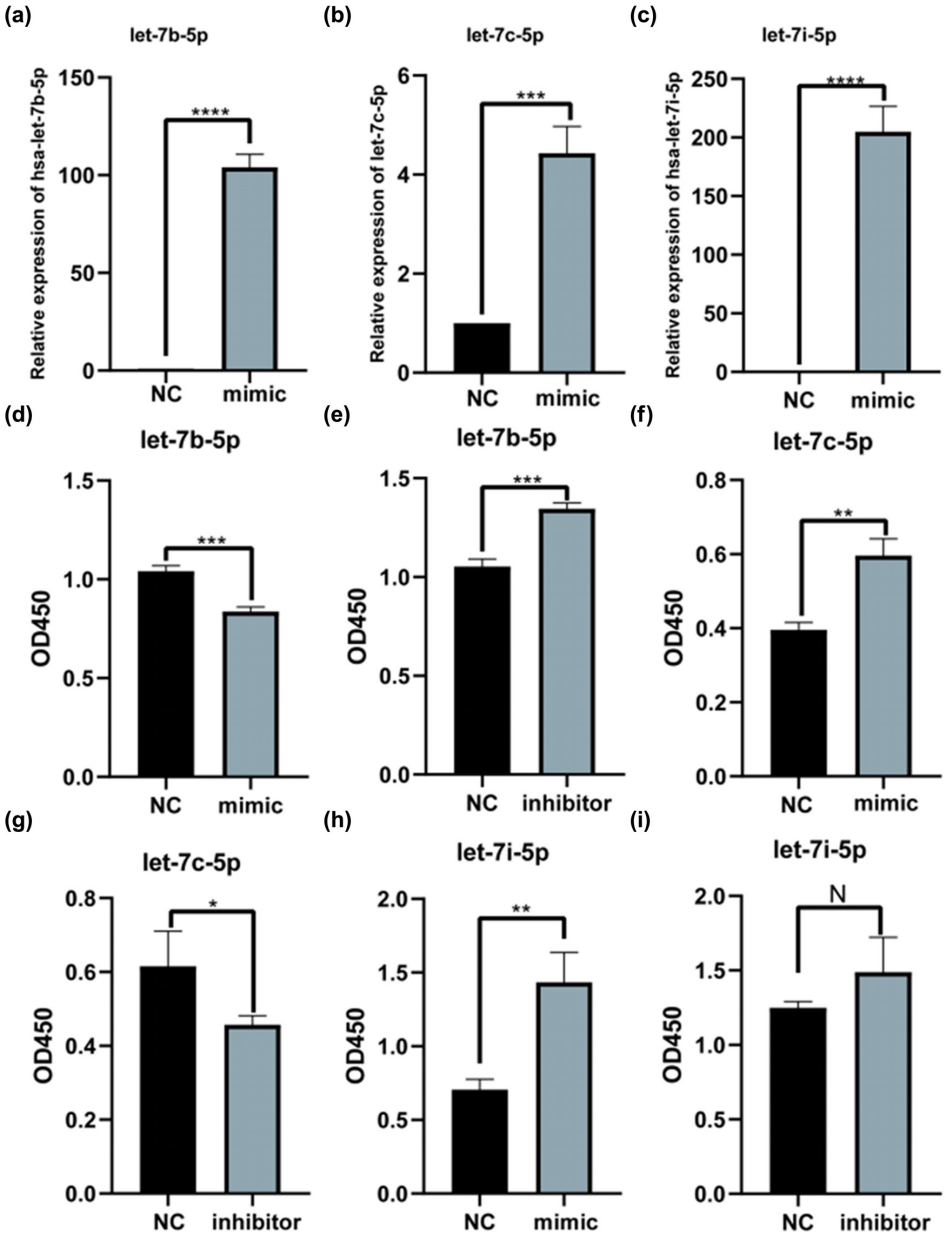
Let-7 family regulates the proliferation of HaCaT cells. (a–c) Following transfection of HaCaT for 48 h with the let-7 family members mimic (50 nmol/l) or mimic NC (50 nmol/l), the levels of let-7 family members were detected by qRT-PCR to verify the transfection. (d) HaCaT cells were transfected and proliferated with let-7b-5p mimic. (e) HaCaT cells were transfected and proliferated with let-7b-5p inhibitor. (f) HaCaT cells were transfected and proliferated with let-7c-5p mimic. (g) HaCaT cells were transfected and proliferated with let-7c-5p inhibitor. (h) HaCaT cells were transfected and proliferated with let-7i-5p mimic. (i) HaCaT cells were transfected and proliferated with let-7i-5p inhibitor. The viability of HaCaT cells was proliferation using CCK-8 assays. *P < 0.05, ***P < 0.001.

To explore the role of the let-7 family in the skin, we transfected let-7b-5p, let-7c-5p, and let-7i-5p into HaCaT cells. A let-7b-5p, let-7c-5p, or let-7i-5p mimic was added to the HaCaT cell culture. Transfection with let-7b-5p, let-7c-5p, or let-7i-5p mimic considerably increased let-7b-5p, let-7c-5p, and let-7i-5p expression levels in HaCaT cells, respectively (Figure 4a–c). Additionally, cell proliferation was inhibited at 48 h after transfecting cells with the let-7c-5p mimic (Figure 4d). HaCaT cell proliferation was enhanced 48 h post-transfection with the let-7c-5p inhibitor (Figure 4e). Cells transfected with let-7c-5p mimic (Figure 4f) or let-7i-5p mimic (Figure 4h) showed higher HaCaT cell proliferation. In addition, HaCaT cell proliferation was inhibited when the expression of let-7c-5p (Figure 4g) or let-7i-5p was inhibited (Figure 4i).

### 3.4 Let-7 family affects HaCaT cell apoptosis

**Figure 5:**
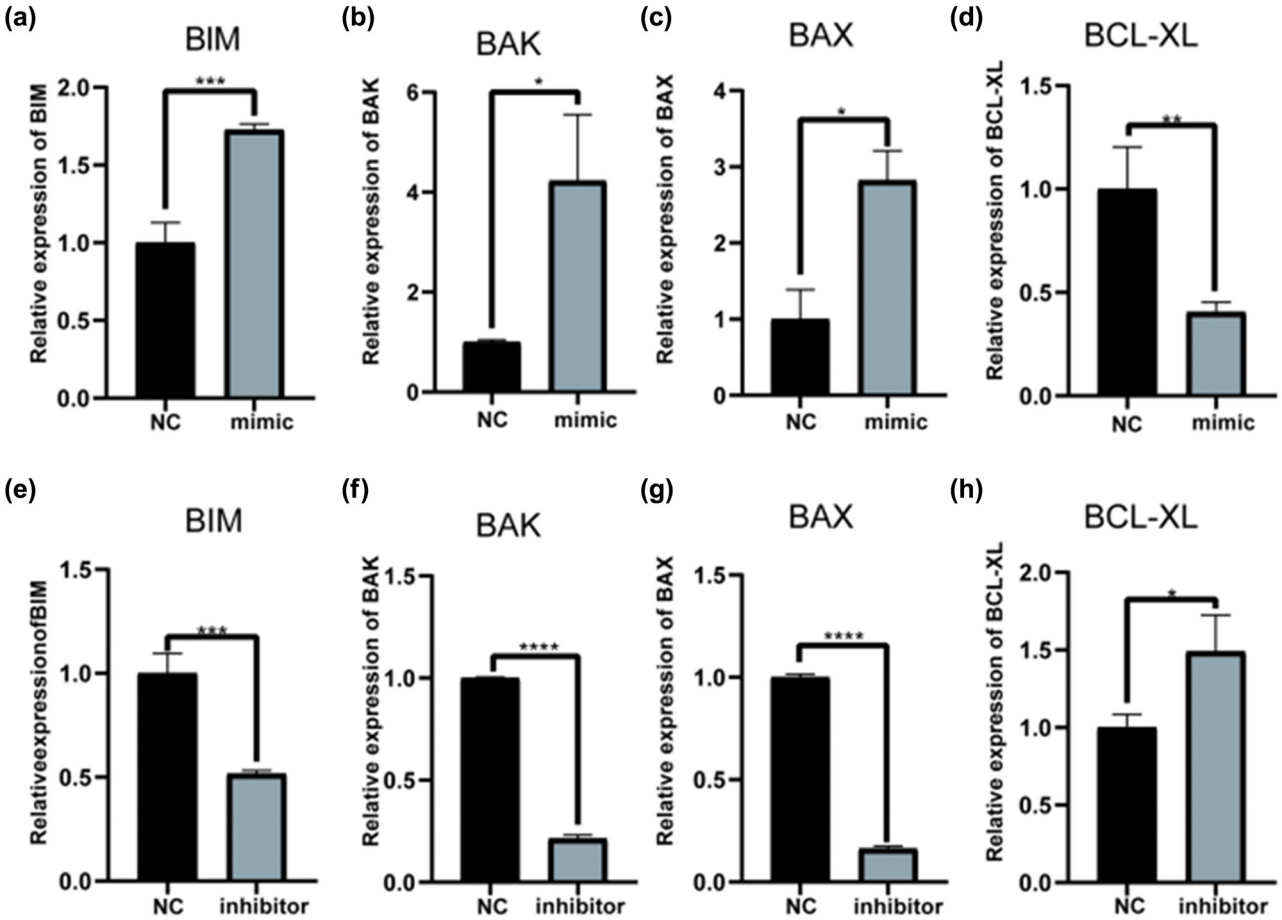
Let-7b-5p regulates the expression of apoptosis-related mRNA in HaCaT cells. (a) BIM, (b) BAK, (c) BAX, and (d) BCL-XL mRNA expression was detected by qRT-PCR after 48 h after transfected of let-7b-5p mimic in HaCaT cells. At 48 h after the HaCaT cells being transfected with the three different concentrations of let-7b-5p inhibitor, (e) BIM, (f) BAK, (g) BAX, and (h) BCL-XL mRNA expression was detected by qRT-PCR. *P < 0.05, ***P < 0.001 vs NC.

**Figure 6:**
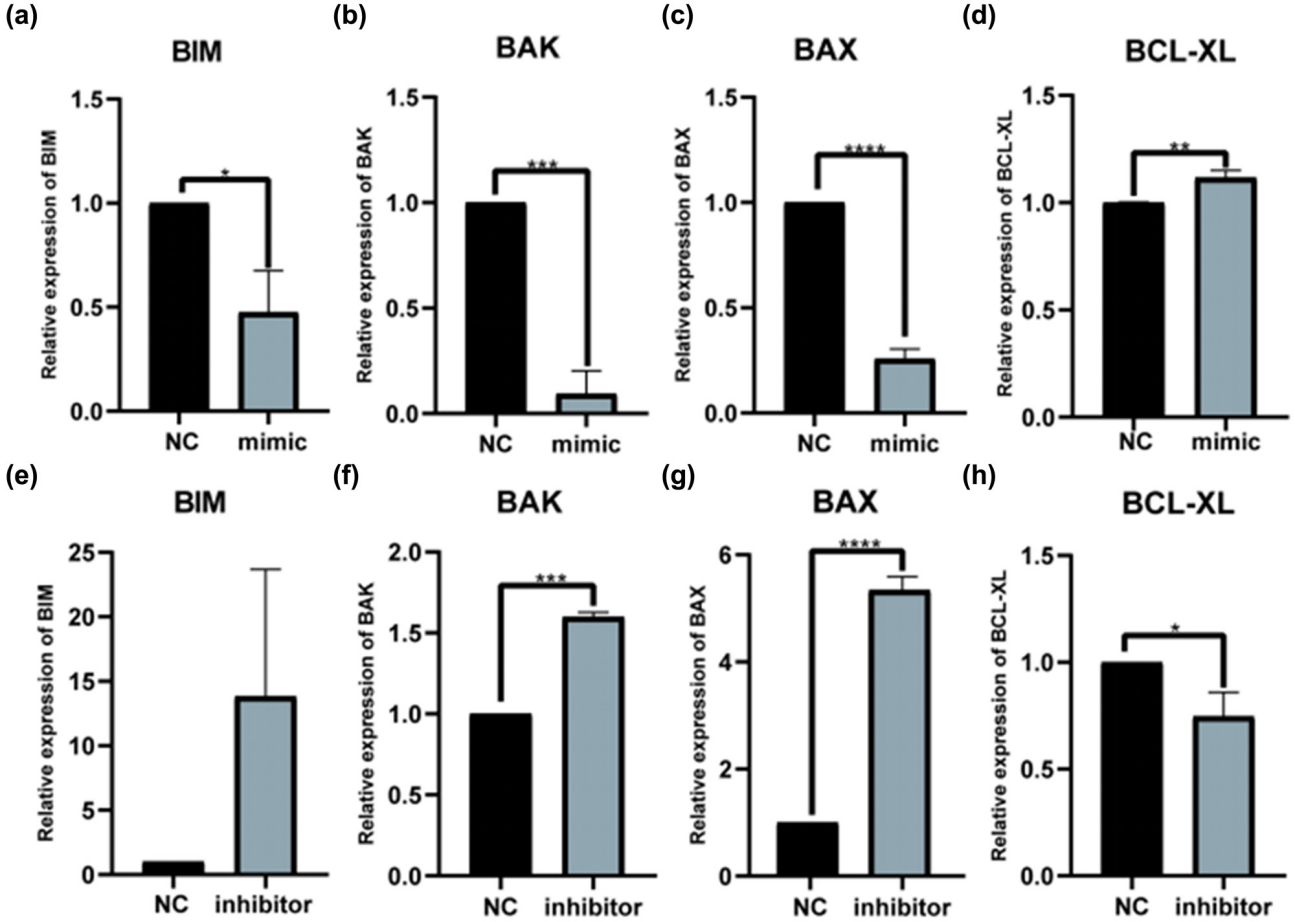
Let-7c-5p regulates the expression of apoptosis-related mRNA in HaCaT cells. (a) BIM, (b) BAK, (c) BAX, and (d) BCL-XL mRNA expression was detected by qRT-PCR after 48 h after transfected of let-7c-5p mimic in HaCaT cells. At 48 h after the HaCaT cells being transfected with the three different concentrations of let-7c-5p inhibitor, (e) BIM, (f) BAK, (g) BAX, and (h) BCL-XL mRNA expression was detected by qRT-PCR. *P < 0.05, ***P < 0.001 vs NC.

**Figure A1:**
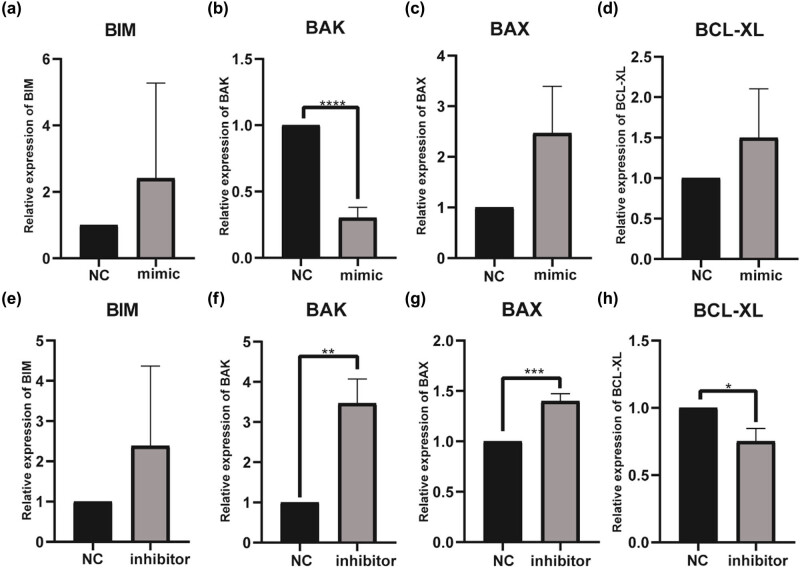
Let-7i-5p regulates the expression of apoptosis-related mRNA in HaCaT cells. (a) BIM, (b) BAK, (c) BAX and (d) BCL-XL mRNA expression was detected by qRT-PCR after 48 hours after transfected of let-7i-5p mimic in HaCaT cells. At 48 h after the HaCaT cells being transfected with the let-7i-5p inhibitor, (e) BIM, (f) BAK, (g) BAX and (h) BCL-XL mRNA expression was detected by qRT-PCR. *P < 0.05, ***P < 0.001 vs NC.

To further investigate whether let-7 could induce apoptosis, subsequently altering HaCaT cell proliferation, we performed qPCR and flow cytometry using HaCaT cells treated with let-7 family mimics and inhibitors for 48 h. The qPCR results showed that let-7b-5p mimic increased *BIM*, *BAK*, and *BAX* expression levels, initiating apoptosis [25] while decreasing *BCL2L1* (*BCL-XL*) expression levels, which have been linked to the suppression of apoptosis [26] (Figure 5a–d). In addition, treatment with the let-7b-5p inhibitor decreased the expression of *BIM*, *BAK*, and *BAX* and increased *BCL-XL* expression (Figure 5e–h). Thus, let-7b-5p was considered to induce apoptosis in HaCaT cells. Let-7c-5p overexpression decreased *BIM*, *BAK*, and *BAX* expression and increased *BCL-XL* expression levels (Figure 6a–d). Treatment with the let-7c-5p inhibitor increased *BAK* and *BAX* expression but decreased the expression of *BCL-XL* (Figure 6e–h). Nevertheless, it has a negligible impact on *BIM* expression. Changes in let-7i-5p expression affected *BAK* expression alone, and no significantly different effects were observed on other apoptotic genes. The results are presented in Figure A1.

**Figure 7:**
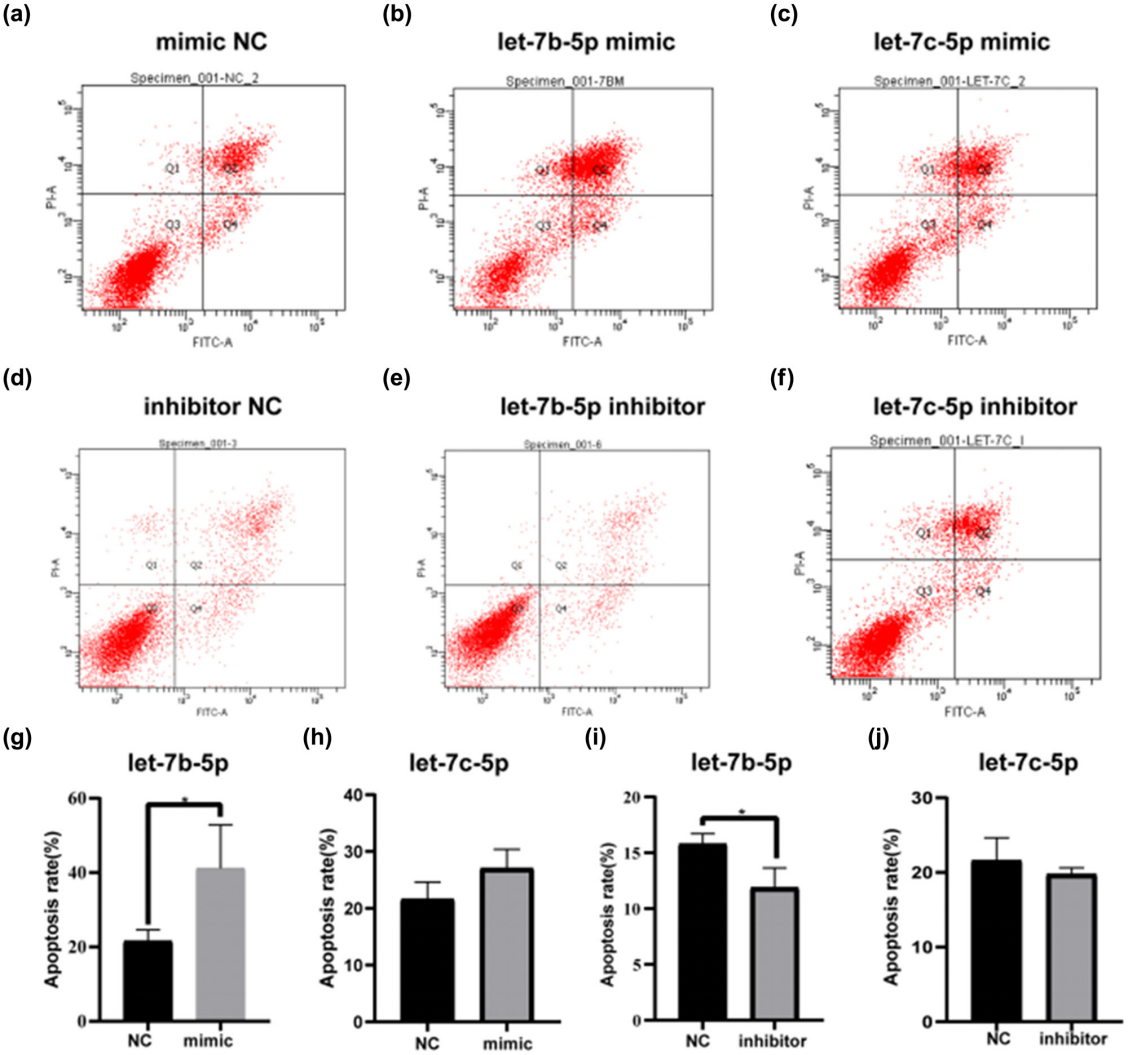
Let-7 family regulates the apoptosis of HaCaT cells. Flow cytometry with annexin V-fluorescein isothiocyanate/propidium iodide double staining detected apoptosis in HaCaT cells after transfected of (a) mimic NC, (b) let-7b-5p mimic, (c) let-7c-5p mimic, (d) inhibitor NC, (e) let-7b-5p inhibitor, and (f) let-7c-5p inhibitor for 48 h. (g–j) Statistical diagram of the results of flow cytometry independent experiments. *P < 0.05, ***P < 0.001 vs NC.

Flow cytometry showed that the let-7b-5p mimic promoted HaCaT cell apoptosis (Figure 7b and g), and treatment with let-7b-5p inhibitor delayed HaCaT cell apoptosis (Figure 7e and i). Unlike the control group, we observed a very significant response to both treatments in the experimental group. Let-7c-5p treatment showed the same apoptosis-promoting effect; nevertheless, HaCaT apoptosis was not statistically significantly different (Figure 7h and j).

Based on these data, we noted that let-7b-5p mimics induced apoptosis and reduced the HaCaT cell proliferation. In contrast, let-7b-5p inhibitors inhibited apoptosis and enhanced HaCaT cell proliferation; statistically significant differences were observed among all the tests mentioned earlier. Therefore, let-7b-5p was used as an ideal *in vitro* regulator in subsequent experiments.

### 3.5 Let-7b-5p regulates ΔNp63 expression in HaCaT cells

**Figure 8:**
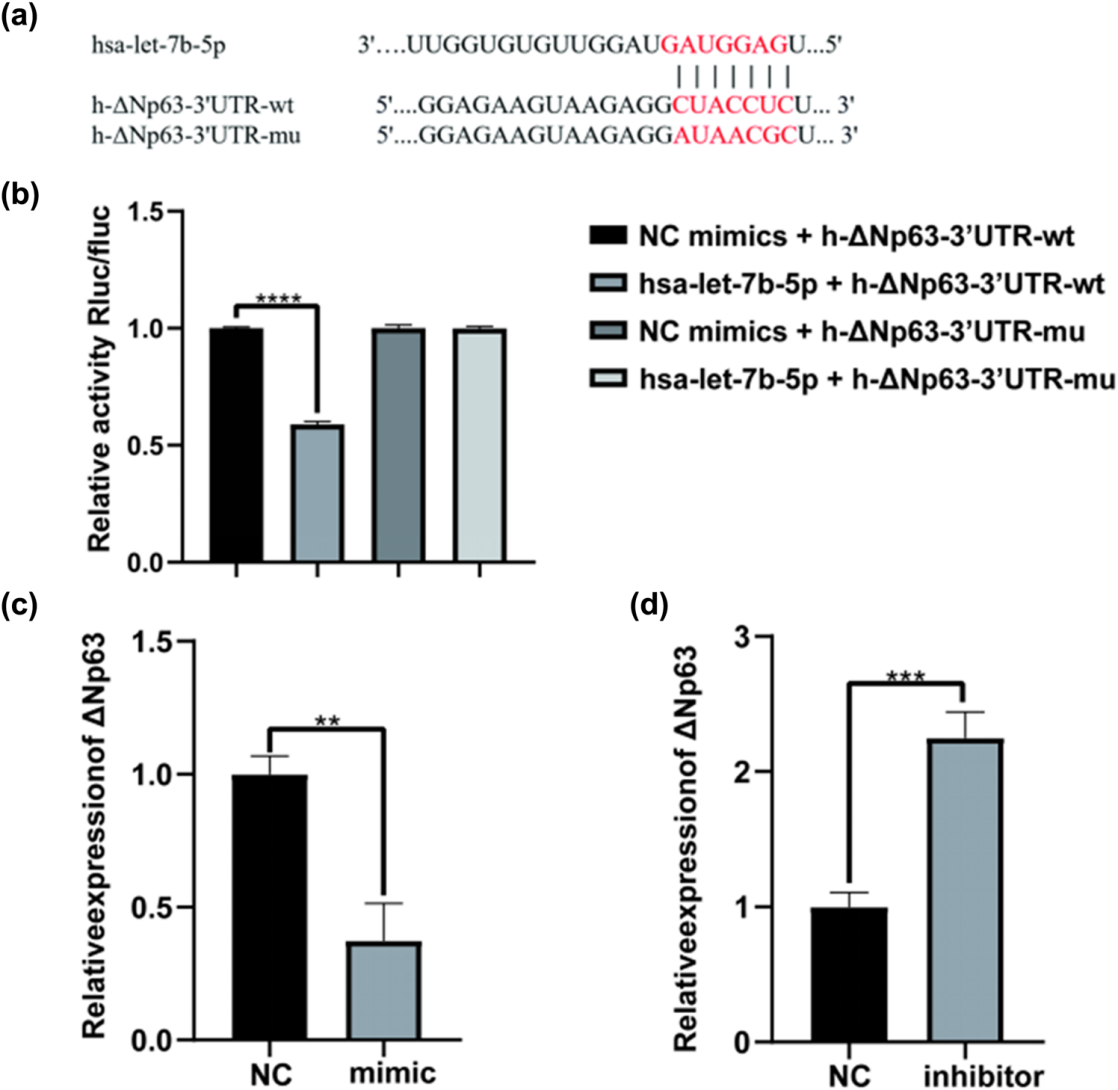
Let-7b-5p regulates the expression of ΔNp63 in HaCaT cells. (a) The online RNAhybrid 2.2 software (https://bibiserv.cebitec.uni-bielefeld.de/rnahybrid/) indicated that let-7b-5p is potentially bound to ΔNp63 mRNA. (b) Dual-luciferase reporter gene system assay was performed to validate the binding sites of let-7b-5p with ΔNp63. (c and d) let-7b-5p regulates ΔNp63 mRNA expression was detected by qRT-PCR. *P < 0.05, ***P < 0.001.

Based on the aforementioned results, we predicted let-7b-5p target genes via TargetScan and identified ΔNp63 among the predicted target genes. RNAhybrid 2.2 website analysis predicted that let-7b-5p could bind to the 3′-UTR sequence of ΔNp63 (Figure 8a). ΔNp63 is a regulatory gene crucial for normal skin development [27–29]. As a result, it is plausible to speculate that let-7b-5p may affect HaCaT cells by regulating ΔNp63 expression. Therefore, we investigated the inter-regulation of let-7b-5p and ΔNp63.

A double luciferin reporter assay was performed to identify functional interactions between let-7b-5p and its target gene ΔNp63 and identify the binding location for let-7b-5p and ΔNp63 3′-UTR. We found that let-7b-5p had no significant regulatory effect on ΔNp63 3′-UTR-MT expression; however, ΔNp63 3′-UTR-WT expression was significantly suppressed. This result indicates that let-7b-5p can bind to the 3′-UTR region of ΔNp63 (Figure 8b). Furthermore, the qPCR analysis showed that mock transfection of let-7b-5p decreased ΔNp63 expression (Figure 8c), whereas the exact opposite result occurred when the let-7b-5p expression was repressed (Figure 8d). Taken together, let-7b-5p downregulated ΔNp63 expression by binding to the 3′-UTR region of ΔNp63.

### 3.6 Let-7b-5p downregulates ΔNp63 expression affecting HaCaT cell proliferation and apoptosis

**Figure 9:**
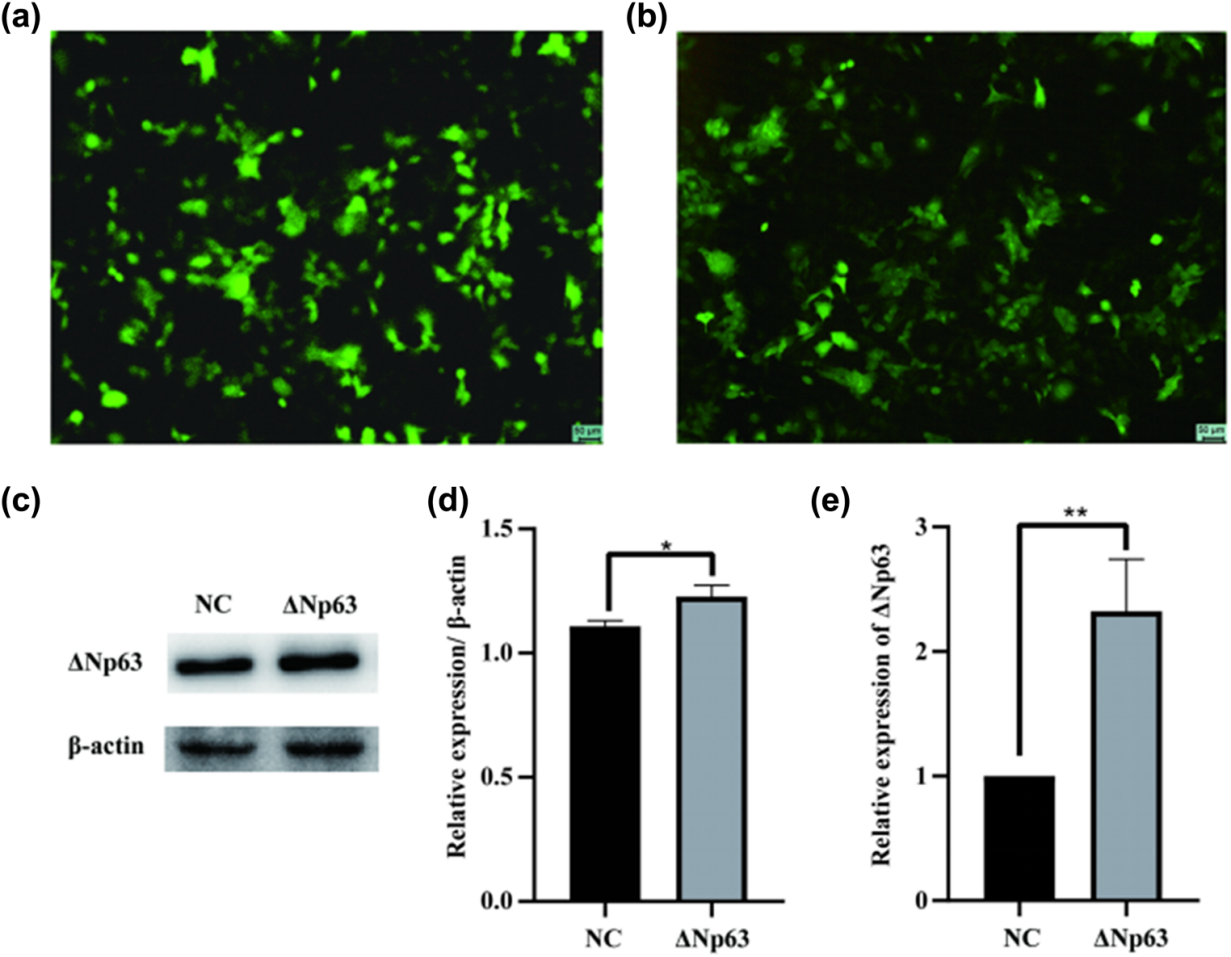
Detection of the enhancement effect of ΔNp63 in HaCaT cells. Virus vector infection renderings, the vector comes with green fluorescence: (a) negative control lentivirus (Lv-NC), (b) ΔNp63 overexpression lentivirus (Lv-ΔNp63), (c) western blot assay was used to evaluate the enhancement effect of ΔNp63 in HaCaT cells, (d) column gram of western blot assay results, and (e) stable ΔNp63-overexpressed cell line was successfully established in HaCaT cells by infecting with Lv-ΔNp63 or Lv-NC, respectively, through RT-qPCR assay. *P < 0.05, ***P < 0.001.

For the purpose of determining if the regulatory effect of let-7b-5p on HaCaT cells was related to ΔNp63 expression, we constructed a ΔNp63 overexpression lentiviral vector, and a ΔNp63 overexpression model was established in HaCaT cells by transfecting the ΔNp63 overexpression lentivirus (Figure 9). Western blotting and qPCR assays confirmed that ΔNp63 was upregulated in cells infected with the ΔNp63 overexpression lentivirus (Lv-ΔNp63) compared to that in cells infected with the negative control lentivirus (Lv-NC).

**Figure 10:**
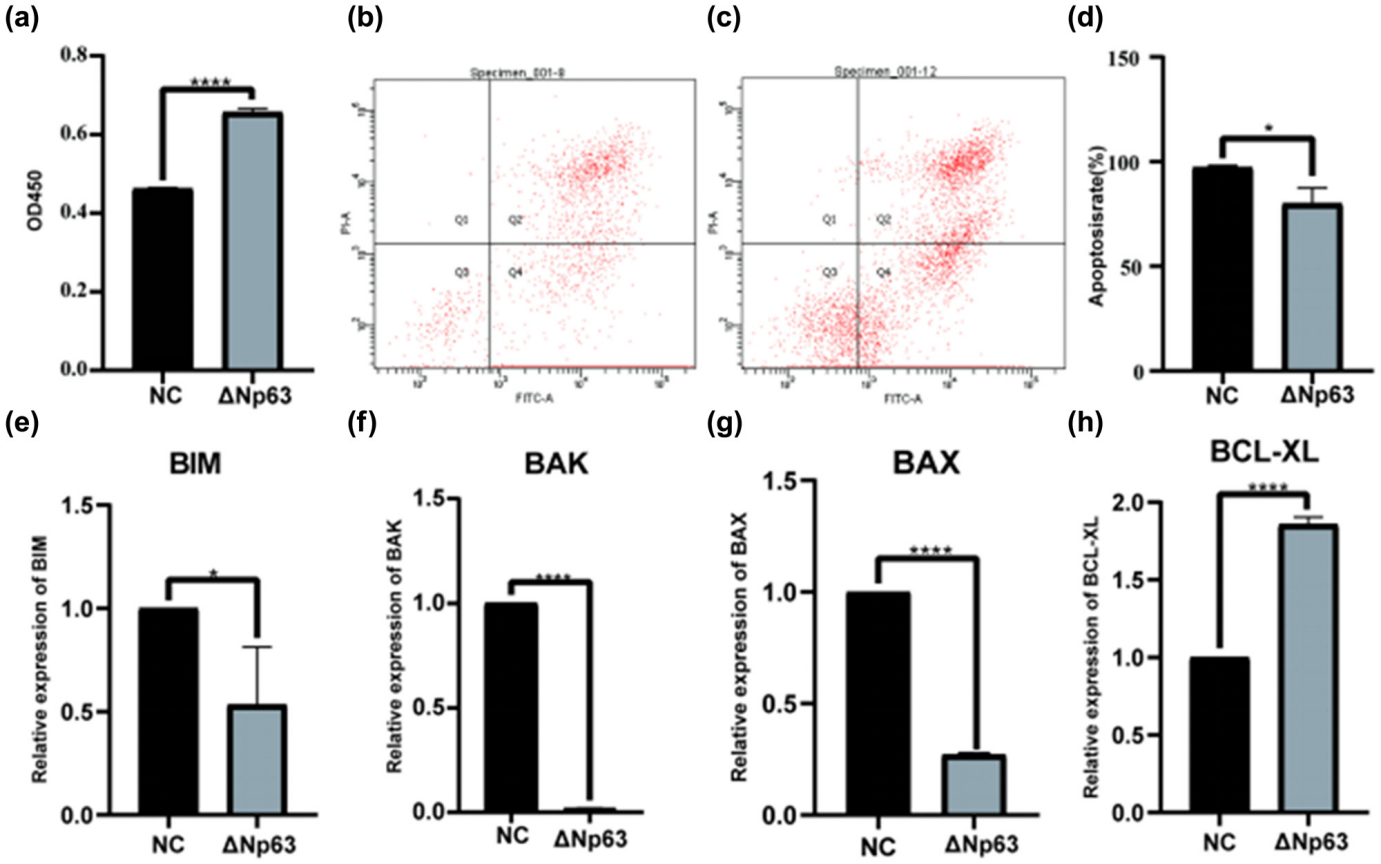
ΔNp63 reversed the effect of let-7b-5p on HaCaT cells proliferation and apoptosis. (a) The proliferation effect of ΔNp63 overexpression on HaCaT cells was detected by CCK-8 assays; flow cytometry with annexin V-fluorescein isothiocyanate/propidium iodide double staining detected apoptosis in HaCaT cells after infection of (b) negative control lentivirus (Lv-NC) or (c) ΔNp63 overexpression lentivirus (Lv-ΔNp63). (d) Statistical diagram of the results of flow cytometry independent experiments; after the HaCaT cells being infected with the Lv-ΔNp63 or Lv-NC, (e) BIM, (f) BAK, (g) BAX, and (h) BCL-XL mRNA expression was detected by qRT-PCR. *P < 0.05, ***P < 0.001.

Next, CCK-8 analysis showed that ΔNp63 overexpression promoted HaCaT cell proliferation compared to that in Lv-NC-treated cells (Figure 10a). In addition, flow cytometry revealed that ΔNp63 overexpression could inhibit HaCaT cell apoptosis compared to Lv-NC-treated cells (Figure 10b–d). Moreover, ΔNp63 overexpression downregulated *BIM*, *BAK*, and *BAX* expression, promoting apoptosis, while upregulating *BCL-XL* expression, ultimately suppressing apoptosis (Figure 10e–h). Thus, ΔNp63 overexpression enabled let-7b-5p to exert a reversal effect on HaCaT cell proliferation and apoptosis.

### 3.7 Let-7b-5p affects the PI3K-AKT signaling pathway

**Figure 11:**
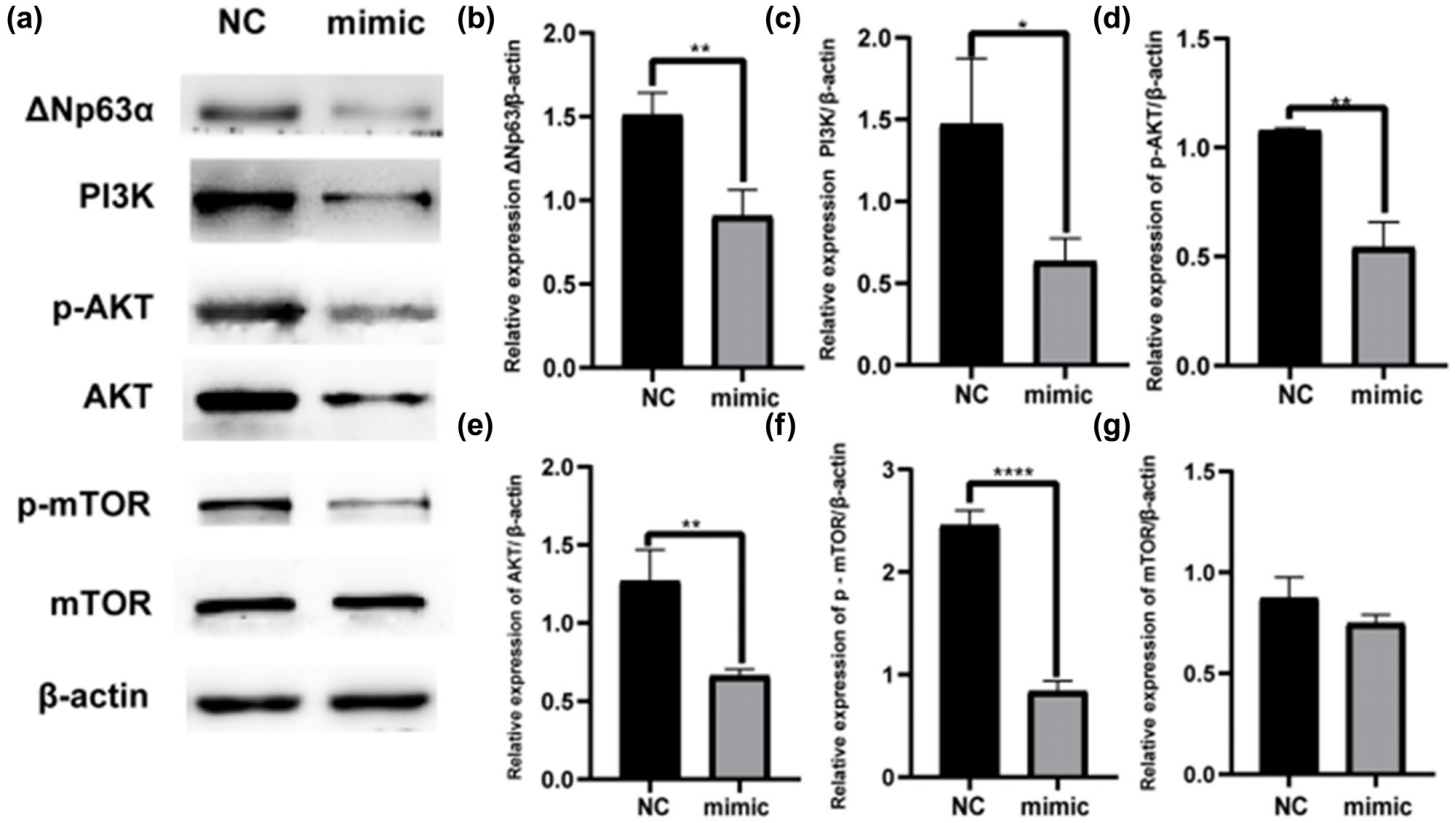
Western blotting analysis indicated the levels of proteins. (a) The western blotting assay indicated that the levels of proteins ΔNp63, PI3K, AKT, p-AKT, mTOR and p- mTOR in the let-7b-5p mimic group were significantly lower than that in the NC group. (b–g) The relative expression was calculated via normalized to β-actin expression. *P < 0.05, ***P < 0.001.

Based on western blotting results (Figure 11), ΔNp63 expression in the let-7b-5p mimic group was significantly reduced. Furthermore, KEGG pathway enrichment results revealed that skin development-related DEmiRNAs might affect the PI3K/AKT pathway. Western blotting led to the conclusion that the increase in let-7b-5p expression decreased the total PI3K, AKT, p-PI3K, p-AKT, and p-mTOR protein expression but had no effect on the total mTOR.

**Figure 12:**
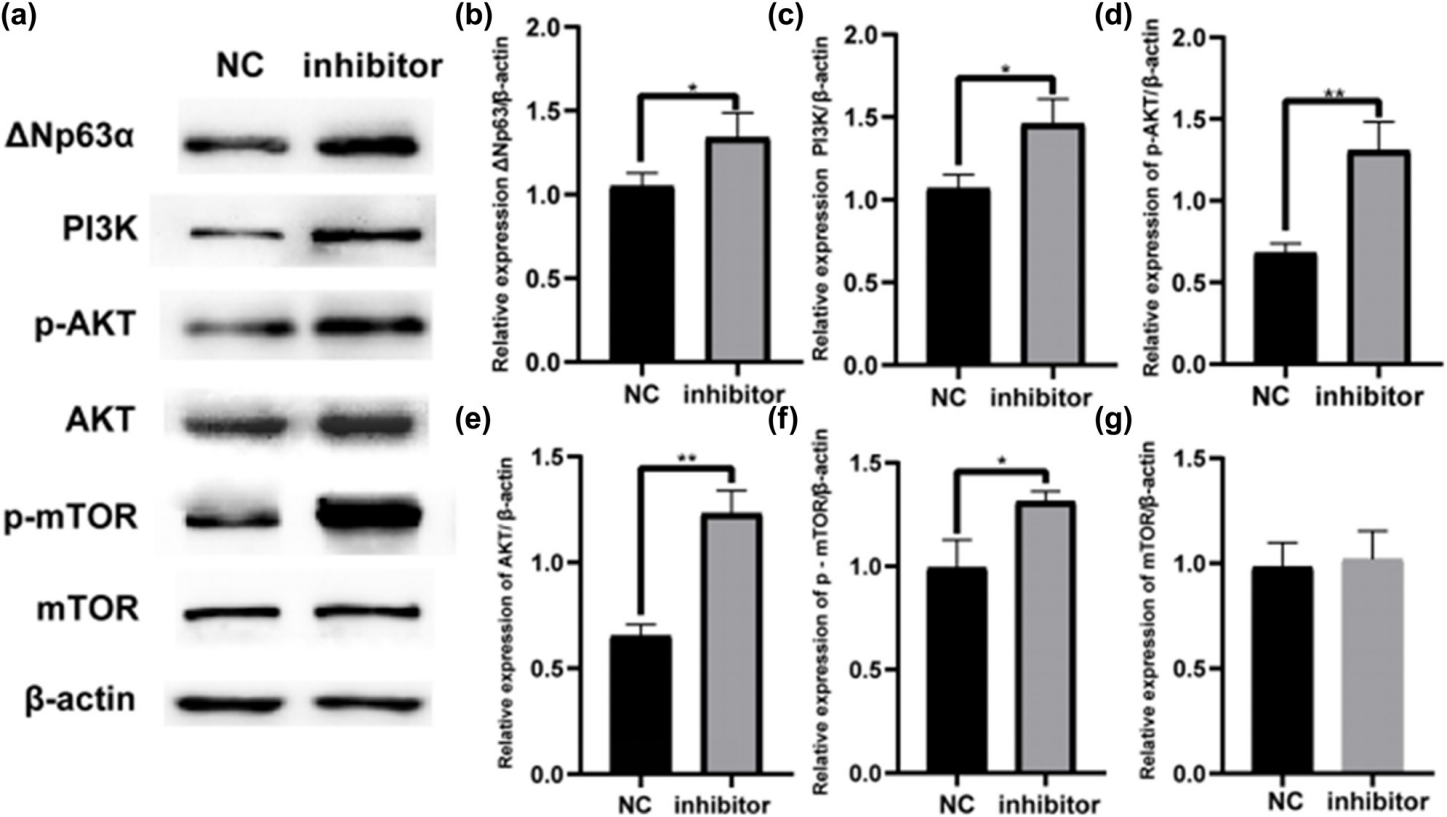
Western blotting analysis indicated the levels of proteins. (a) The western blotting assay indicated that the levels of proteins ΔNp63, PI3K, AKT, p-AKT, mTOR, and p- mTOR in the let-7b-5p inhibitor group were significantly higher than that in the NC group. (b–g) The relative expression was calculated via normalized to β-actin expression. *P < 0.05, ***P < 0.001.

Western blotting analysis revealed that ΔNp63 expression was significantly increased in the let-7b-5p inhibitor group (Figure 12). The total PI3K, AKT, p-PI3K, p-AKT, and p-mTOR protein expression levels increased, and the PI3K/AKT pathway was activated. The let-7b-5p inhibition group showed no difference in terms of total mTOR protein expression.

**Figure 13:**
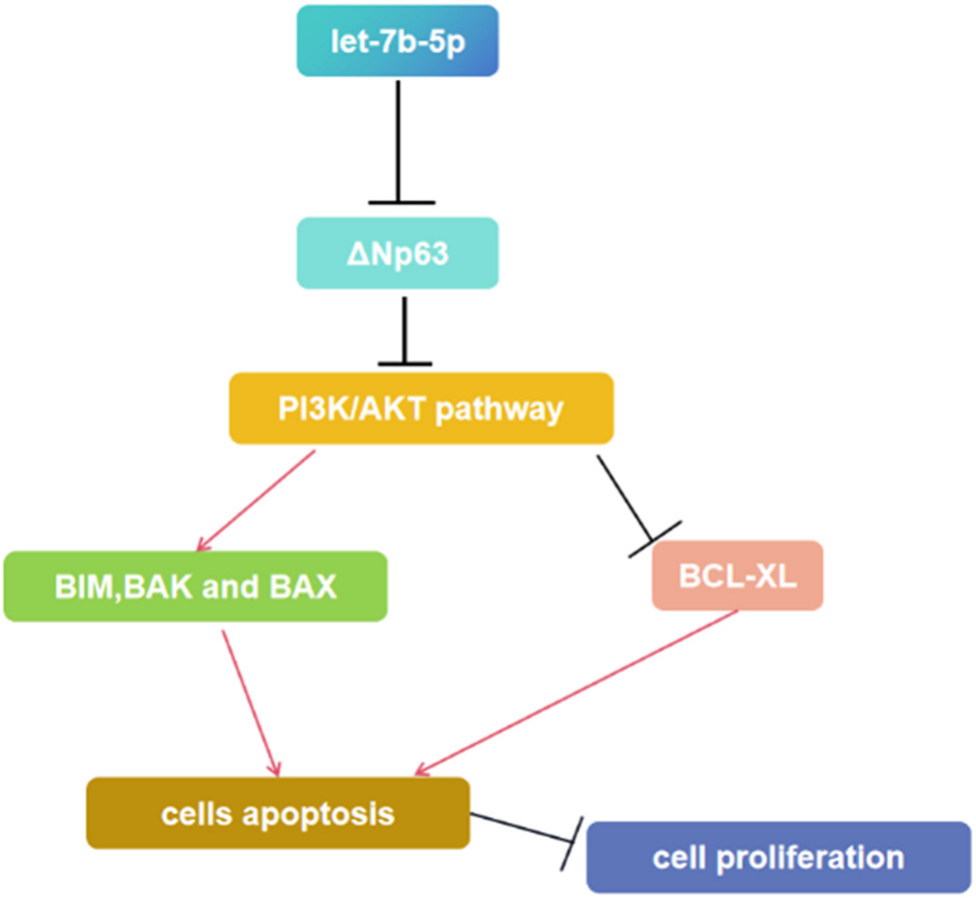
The schematic showing the possible signaling of let-7b-5p in skin development. Let-7b-5p inhibits cell proliferation, induces apoptosis through PI3K-AKT signaling.

Therefore, let-7b-5p may downregulate ΔNp63 expression, thereby inducing apoptosis and suppressing HaCaT cell proliferation, possibly via negative regulation of the PI3K/AKT signaling pathway (Figure 13).

## 4 Discussion

The key findings of this study are of vital importance for skin development studies; for instance, the TP63 gene has been found to represent a node gene that regulates skin development [30]. This discovery has fueled skin research development. However, data on the key mechanisms that regulate skin development remain limited. For instance, the miRNAs involved in the regulatory networks that control fetal skin development have not been studied thoroughly. Most published studies have focused on single genes. However, a succession of genes is frequently involved in biological processes, as is the regulation of important genes. Previous studies have shown that ΔNp63 is a pivotal gene associated with skin development, and an improved understanding of the regulatory mechanisms of skin development in the fetus will assist in achieving scar-free wound healing [4]. Therefore, we aimed to identify key miRNA families present in fetal skin samples during development via miRNA sequencing. Then, by predicting and experimentally verifying the relationship between miRNAs and ΔNp63, we intended to know more concerning the regulatory systems that control fetal skin development.

In this study, 363 miRNAs were identified as DEmiRNAs between skin samples associated with <11 and >11 weeks of gestation. According to GO analysis, the function of target mRNAs is primarily connected to transcription regulation and cell proliferation, which play major roles in skin development. Additionally, target mRNAs were predominantly enriched in the PI3K-AKT, mitogen-activated protein kinase (MAPK), and cancer-associated signaling pathways, according to the KEGG pathway analysis. The epidermal growth factor receptor (EGFR)/AKT/PI3K signaling pathway is closely related to cell proliferation and apoptosis [31], whereas the MAPK pathway is associated with apoptosis in HaCaT cells [32]. Despite the recent identification of a few signaling pathways that may control the proliferation and death of epithelial cells, more research is required to determine the precise regulation mechanism of miRNA in skin cells. After performing a functional analysis of the target mRNAs, the TAM 2.0 tool was used to identify the significant miRNA–disease associations of the DEmiRNAs. According to the analysis, the association with melanoma was the most important among all associations. Reports have shown that miR-10b promotes melanoma progression [33], while miR-429 adversely affects melanoma cell proliferation and migration [34], and hsa-let-7b also suppresses melanoma cell proliferation [35]. These findings support our predictions. The findings of this study demonstrated that miRNAs frequently bind to mRNAs to influence their expression. Furthermore, based on the TAM 2.0 analysis, it was noted that upregulated miRNAs were mainly enriched in the let-7 family. The miRNA-98 member of the let-7 family adversely affects the proliferation of human hypertrophic scar fibroblasts [36]. We hypothesized that the let-7 family might represent the main miRNA family involved in fetal skin development. To verify this hypothesis, we selected let-7b-5p, let-7c-5p, and let-7i-5p in the let-7 family for evaluation at the cell level. CCK-8 analysis showed that let-7b-5p inhibited HaCaT cell proliferation, whereas let-7c-5p and let-7i-5p promoted HaCaT cell proliferation. Let-7b-5p enhanced apoptosis in HaCaT cells via controlling the expression of apoptosis regulators including BCL-2 family members, as demonstrated by qPCR and flow cytometry experiments. These experimental findings demonstrated that let-7b-5p controlled apoptosis and proliferation in HaCaT cells.

Next, we investigated how let-7b-5p functions in HaCaT cells. ΔNp63 was identified among the mRNAs that are targets of DEmiRNAs. Previous studies have shown that p63, as the most critical regulator of epidermal development, has a significant impact on the growth and differentiation of HaCaT cells as well as the development of embryonic epidermis. Moreover, the loss of p63 leads to ectodermal dysplasia and normal epidermal tissue development in newborn mice [37]. Similar to its involvement in the initial regulation of skin development, p63 is involved in regulating the regenerative repair process after skin injury in mice [38]. Low levels of heterodimer p73 of p63 result in reduced basement keratinocyte cell proliferation and DNA damage, delaying wound healing [39]. p63 can not only regulate the development of normal skin, but it can also regulate the progression of various skin diseases. Studies have reported that overexpression of the p63 protein is a part of the underlying mechanism in the development of Hay–Wells syndrome [40]. In mice, ZNF185 can act as a target gene of p63 to regulate the dynamic balance of epidermal differentiation and the occurrence of squamous carcinoma [41]. Moreover, p63 can help prevent particular dermatitis brought on by suppression of type 2 cytokines (interleukin 4 [IL-4] and IL-13) linked to keratinocyte development [40]. Furthermore, dysfunctional telomeres can damage epidermal stem cells, influencing skin and hair follicle development by interfering with the BMP/pSmad/p63 signaling pathway [42]. ΔNp63, as the main p63 subtype in the complex squamous epithelium, not only regulates the proliferation and differentiation of normal cells but also promotes cell survival and cell proliferation [43]. ΔNp63 has been confirmed to participate in the regulation of epithelial tissue morphogenesis. For instance, knockout of the isomer ΔNp63γ of ΔNp63 can promote HaCaT cell proliferation and migration and inhibit cellular aging [44]. In contrast, the isomer ΔNp63α of ΔNp63 can activate the transcription of EGFR and promote cell proliferation [45]. Let-7b-5p’s ability to associate to the 3′-UTR region of Np63 was anticipated by RNAhybrid 2.2 online analysis, confirmed via double luciferase reporter assays. This present study showed that let-7b-5p inhibited ΔNp63 expression when it associates to the 3′-UTR region of ΔNp63. When ΔNp63 was upregulated, let-7b-5p exerted the opposite effect on HaCaT cell proliferation and apoptosis. Therefore, our experimental results proved that let-7b-5p’s effect on HaCaT cell proliferation and apoptosis was accomplished by controlling Np63 expression. Furthermore, earlier KEGG predictions indicated that the PI3K-AKT signaling pathway would serve as an important regulatory pathway in skin growth. Western blotting tests were used to determine the impact let-7b-5p would have on the PI3K-AKT signaling pathway’s protein expression. When let-7b-5p expression was inhibited, PI3K-AKT signaling was activated and cell proliferation was initiated. This is consistent with findings of earlier studies, which report that the PI3K-AKT signaling pathway can promote HaCaT cell proliferation [46].

In conclusion, this work highlights the significant influence of the expression of let-7b-5p in skin development. Although let-7 has been shown to inhibit the metastasis of cutaneous melanoma cells [47], its impact on skin that is normal has not yet been studied. The findings of this study indicate that let-7b-5p influences the PI3K-AKT signaling pathway via controlling the expression of Np63, thereby regulating skin cell proliferation and apoptosis. The study findings reveal the potential mechanisms involved in skin development and may provide a new therapeutic target for facilitating scar-free skin healing.

## 5 Limitations

There are some limitations to the results of this study. The small sample size may lead to some bias in the results. This study focused on the regulatory role of let-7b-5p, but miRNAs often have complex multi-target effects in cellular regulation.

## Abbreviations

CCK-8: cell counting kit-8
DEmiRNAs: differentially expressed miRNAs
EGFR: epidermal growth factor receptor
GO: gene ontology
IL: interleukin
KEGG: Kyoto Encyclopedia of Genes and Genomes
Lv-NC: negative control lentivirus
Lv-ΔNp63: ΔNp63 overexpression lentivirus
MAPK: mitogen-activated protein kinase
miRNAs: microRNAs
mTOR: mechanistic target of rapamycin
MT: mutant
NC: negative control
PCA: principal component analysis
PI3K: phosphoinositide 3-kinase
p-mTOR: phosphorylated mTOR
qPCR: quantitative real-time PCR
UTR: untranslated region
WT: wild-type
